# l-Carnosine loaded on carboxymethyl cellulose hydrogels for promoting wound healing†

**DOI:** 10.1039/d4ra00135d

**Published:** 2024-06-07

**Authors:** Wei Zhang, Xinyi Li, Wenjian Chen, Xiaoyi Huang, Tianfeng Hua, Jinpeng Hu, Jing Zhu, Sheng Ye, Xiaojing Li

**Affiliations:** a Department of Plastic Surgery, The First Affiliated Hospital of Anhui Medical University Hefei 230022 Anhui China; b School of Materials and Chemistry, Anhui Agricultural University Hefei Anhui 230036 China; c Department of Emergency Surgery & the 2nd Department of Intensive Care Unit, The Second Hospital of Anhui Medical University Hefei Anhui 230001 China; d Department of Orthopaedics, Anhui Provincial Children's Hospital Hefei Anhui 230022 China

## Abstract

Wound management remains a challenge in clinical practice. Nowadays, patients have an increasing demand for wound repair with enhanced speed and quality; therefore, there is a great need to seek therapeutic strategies that can promote rapid and effective wound healing. In this study, we developed a carboxymethyl cellulose hydrogel loaded with l-carnosine (CRN@hydrogel) for potential application as a wound dressing. *In vitro* experiments confirmed that CRN@hydrogel can release over 80% of the drug within 48 h and demonstrated its favorable cytocompatibility and blood compatibility, thus establishing its applicability for safe utilization in clinical practice. Using a rat model, we found that this hydrogel could promote and accelerate wound healing more effectively. These results indicate that the novel hydrogel can serve as an efficient therapeutic strategy for wound treatment.

## Abstract 2

A novel carboxymethyl cellulose hydrogel loaded with l-carnosine exhibits remarkable efficacy in enhancing the wound-healing process.

## Graphical abstract

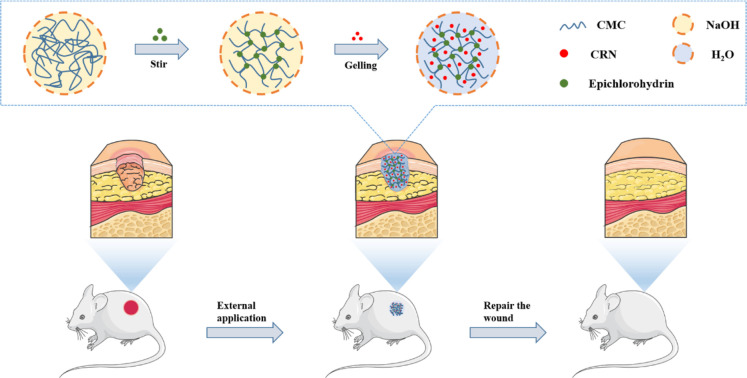

## 1. Introduction

Wounds caused by daily activities require prompt treatment because the injured skin's barrier function against infection is impaired,^1^ and if the injured skin is left untreated, external bacterial infection may occur and potentially lead to serious complications and may even endanger life.^2,3^ Wound healing is a timely and orderly sequential process. It involves the organism recovering its tissue and functional integrity after tissue damage and encompasses inflammatory, proliferative, and remodeling stages.^4,5^ Imbalances occurring at any stage of wound healing can potentially result in adverse outcomes, such as the development of chronic non-healing wounds or the formation of pathological scars.^6^ Currently, there is a growing demand among patients for both speedy and high-quality wound healing. Therefore, there is an urgent need to explore treatment strategies that can promote the rapid and high-quality healing of wounds.

Traditional wound dressings, such as gauzes, tapes, and bandages, have some inherent structural and functional limitations. This results in their inadequate ability to stimulate hemostasis, block microbial infections, and speed up the wound healing process.^7^ Hydrogels, characterized by three-dimensional network structures composed of chemically or physically crosslinked polymers, are increasingly regarded by scholars as the optimal choice for wound management.^8–10^ Hydrogels can maintain a profoundly moist environment, simulate the extracellular matrix (ECM), and provide a protective barrier against bacterial and microbial intrusion.^11–13^ Hydrogels have been widely studied and applied in biomedical fields such as tissue engineering, wound dressing, and drug delivery.^14–17^ Their commendable biocompatibility, predictable degradation kinetics, and adjustable mechanical properties render them outstanding candidates for applications such as wound dressings.^18–20^ Owing to the unique structure and high water content of hydrogels, they can create a physical barrier that restricts the movement of bacteria. This barrier prevents bacteria from reaching the wound site. Bacteria require a certain level of humidity for proliferation, but excessively high humidity can also impact their growth. Hydrogels with a high water content can thus limit the growth of bacteria by keeping the environment sufficiently moist.^21–23^ Furthermore, hydrogels can simulate the ECM of human skin tissue, which plays a pivotal role in supporting cellular growth and promoting tissue regeneration.^24,25^ Remarkably, hydrogels can alleviate wound pain by employing evaporative cooling. Additionally, their facile dislodgement from the wound site mitigates the discomfort commonly associated with the removal of conventional dressings.^26–28^ The unique structure of hydrogels means drugs can be easily loaded into the hydrogel matrix and can diffuse through the hydrogel network, resulting in sustained drug release and prolonged therapeutic effects.^29,30^ Carboxymethyl cellulose (CMC), a crucial polysaccharide, represents a chemically modified derivative of cellulose and is recognized as an FDA-approved material.^31,32^ In this regard, CMC hydrogels merit particular attention due to their abundance, non-toxicity, non-immunogenicity, biodegradability, pliability, transparency, and cost-effectiveness.^33,34^ They have formed the foundation for numerous pharmaceuticals and materials that are introduced into the human body. Furthermore, the heightened hydrophilicity and substantial water sorption capacity demonstrated by CMC hydrogels facilitate the absorption of wound exudates. This contributes to the creation of a moist environment around the wound, which is a crucial factor in facilitating the progression of the wound-healing process.^35^ Due to these benefits, CMC hydrogels are extensively employed in the fields of wound dressings and drug delivery.^36,37^

l-Carnosine (CRN) is a kind of natural dipeptide biosynthesized from β-alanine and l-histidine, which is found in various human tissues, such as muscle and the brain.^38^ This dipeptide has been reported to possess various properties, such as anti-inflammatory, antioxidant, and antiglycation effects.^39,40^ Additionally, it is known to be a potent cytosolic buffering agent and chelator of heavy metals.^41^ This endogenous antioxidant has been shown to play a positive role in the prevention and treatment of ischemic and hypoxic diseases in tissues, such as in the kidney, liver, and the brain.^42–44^ Recent studies have provided evidence for the beneficial effects of CRN in wound management.^45^ CRN exhibits the potential to expedite the wound-regeneration process by enhancing the rates of re-epithelization, angiogenesis, and granulation formation.^46^ Histidine, acting as a precursor for histamine synthesis, is acknowledged for its ability to elevate hydroxyproline levels within granulation tissue. Meanwhile, β-alanine enhances collagen deposition in the wounded region.^47^ Ansurudeen *et al.* found that this endogenous antioxidant accelerated diabetic wound repair, which was associated with an increased expression of the related growth factors and cytokines.^48^ Using a rat model, Roberts *et al.* found that CRN promoted skin wound repair after surgery when given as part of complete enteral nutrition.^49^

**Scheme 1:**
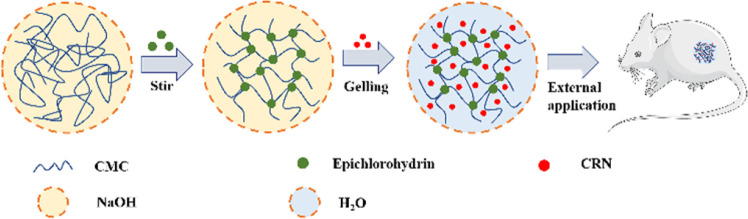
Schematic description of CRN@hydrogel for wound healing.

To the best of our knowledge, there have been no prior investigations into the combined use of CMC hydrogel and CRN for wound management. In this study, we harnessed the distinctive properties of the CMC hydrogel and CRN to develop a novel CRN-loaded CMC hydrogel (CRN@hydrogel) (Scheme 1). We also verified its rheology property, release profile, swelling behavior and safety by *in vitro* experiments and its positive role in wound healing by rat model experiments with the aim of providing a new idea and useful reference for the research and development of novel wound-repair materials.

## 2. Results and discussion

### 2.1 Synthesis and characterization of the CMC hydrogel and CRN@hydrogel

**Fig. 1:**
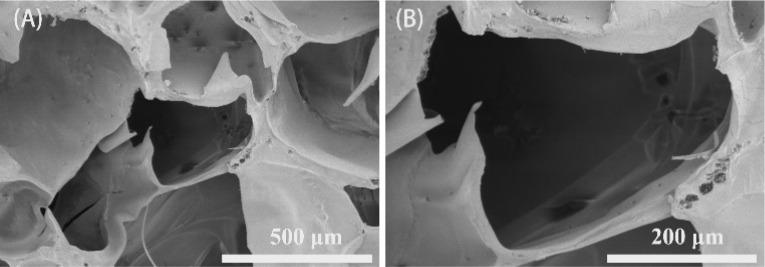
SEM images at different magnifications of the CMC hydrogel. (A) Image at 100 magnification, (B) image at 250 magnification.

To obtain the CMC hydrogel, 3 g of CMC were dissolved in 3% W/V NaOH solution and stirred for 30 min. Then the epichlorohydrin solution was added and stirring was continued to obtain a clear gel precursor. The hydrogel prepolymer was collected and heated for 2 h, and then dried for 24 h to obtain a white and dry gel. The collected dry gel was soaked in water to carry out water adsorption swelling to open the holes in the gel. After freeze-drying, the transparent CMC hydrogel was formed. The morphology of the freeze-dried CMC hydrogel was observed by scanning electron microscopy (SEM). The CMC hydrogel showed porous and interconnected structures (Fig. 1). The pore size of the hydrogel was 463.87 ± 127.62 μm, significantly larger than the pore sizes reported for some hydrogels in previous studies.^7,50,51^ This unique network structure enables the CMC hydrogel to exhibit enhanced capabilities in solvent absorption, drug loading, and drug delivery.

We investigated various ratios of CMC hydrogel and CRN solution. As depicted in Fig. S1,† the hydrogel samples with various ratios all exhibited a transparent gel-like appearance. Upon inverting the test tubes, the occurrence of gel slipping was observed in the samples formed by a mixture of 5 mg and 6 mg CMC hydrogel with 200 μL CRN solution. This observation indicated a lower degree of gelation. In contrast, the sample formed by a mixture of 7 mg CMC hydrogel with 200 μL CRN solution did not exhibit gel slipping, suggesting a superior gelation degree. Therefore, this particular ratio was chosen for the subsequent experiments. The reason for this result may be that when more CMC hydrogel was added, more hydrogen bonds could be formed with water and CRN. This led to a higher degree of crosslinking in the CRN@hydrogel, thereby facilitating improved gelation.

**Fig. 2:**
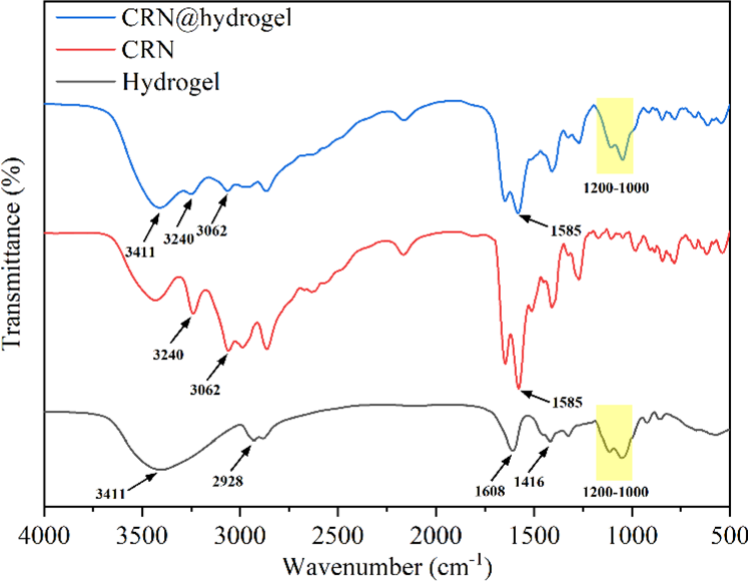
FTIR spectra of CRN, CMC hydrogel, and CRN@hydrogel.

In Fig. 2, the Fourier transform infrared (FTIR) spectrum of the CMC hydrogel showed a broad band at 3411 cm^−1^, which could be related to the stretching of –OH groups and intermolecular and intramolecular hydrogen interactions.^52,53^ Also, bands appeared at 1416 and 1608 cm^−1^, which were attributed to the symmetric and asymmetric stretching vibrations of the carboxylate groups, while a C–H stretching band appeared at 2928 cm^−1^.^52^ The presence of carboxylate groups enables the CMC hydrogel to engage in hydrogen bonding reactions with other compounds. Also, bands were observed in the 1000–1200 cm^−1^ region that were assigned to the C–O and C–C stretching vibration, indicating the polysaccharide structure of the CMC hydrogel.^54^ In the spectrum of free CRN, the peaks related to the terminal cationic amine (NH^3+^) were detected at 3240 and 3062 cm^−1^.^55,56^ Also, asymmetric stretching of the carboxylate group at 1585 cm^−1^ was observed.^57^ The presence of NH^3+^ and carboxylate group in CRN enables it to form hydrogen bonds with other compounds. In the FTIR spectrum of the CRN@hydrogel, characteristic peaks of the CMC hydrogel were observed at 3411 and 1000–1200 cm^−1^. The presence of CRN was confirmed by the characteristic peaks of NH^3+^ at 3240 and 3062 cm^−1^ and the characteristic peak of carboxylate group at 1585 cm^−1^. The FTIR spectrum data indicated the successful incorporation of CRN into the CMC hydrogel, where the combination of CRN and the CMC hydrogel may be related to hydrogen bonding interactions.

### 2.2 Rheology property of the CMC hydrogel

**Fig. 3:**
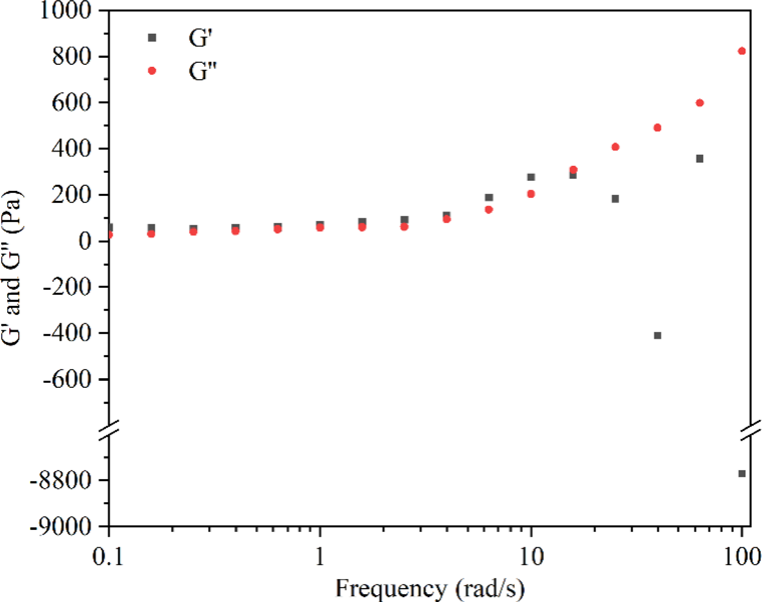
Strain sweep test of the CMC hydrogel.

The rheological experiment results revealed that, during low-frequency shear (<15 rad s^−1^), the storage modulus (*G*′) exceeded the loss modulus (*G*′′), indicating a relatively intact structure of the hydrogel. Conversely, under higher frequency shear (>15 rad s^−1^), *G*′′ exceeded *G*′, and the results for *G*′ exhibited irregular fluctuations (Fig. 3). These findings suggested structural transitions and damage within the hydrogel polymer network.^58^ Following the crossover point, the hydrogel loses its gel-like properties, transitioning into a sol state and becoming non-deformable.^59,60^ Moreover, when the hydrogel was subjected to strains ranging between 10–100%, *G*′ remained greater than *G*′′ (Fig. S2†). These results showed that within this strain range, the internal structure of the hydrogel remained undamaged, maintaining an overall cohesive gel-like state.^61,62^ The advantageous gel structure maintained by the CMC hydrogel is attributed to the presence of hydrogen bonds in their polymer network. In the CMC hydrogel with hydrogen bonds, a higher shear strain is required to disrupt the polymer network.^63^ It is noteworthy that Enoch *et al.* found that an increase in the concentration of CMC could enhance the storage modulus of the hydrogel, making it harder and more stable.^58^ This was attributed to the increase in hydrogen bonds within the polymer network with the elevated concentration of CMC.

### 2.3 Swelling behavior of the CMC hydrogel and CRN@hydrogel

**Fig. 4:**
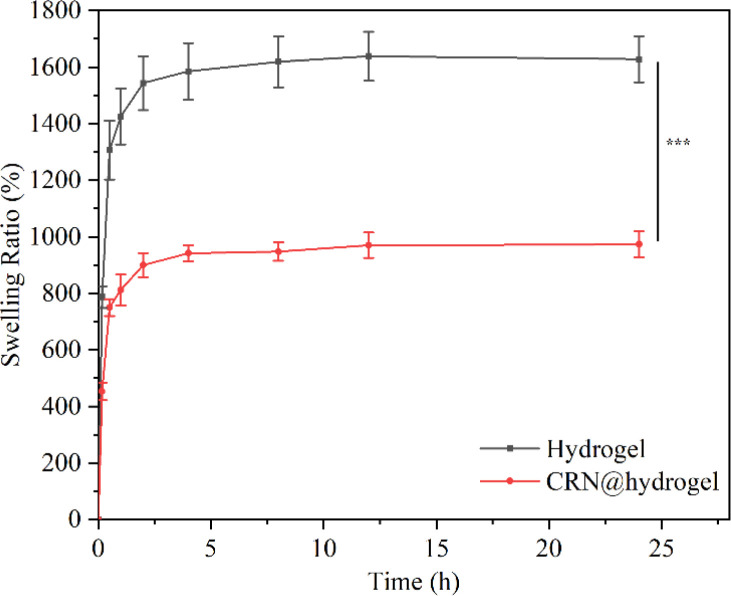
Swelling behavior of the CMC hydrogel and CRN@hydrogel in PBS solution (pH 7.4). ***P < 0.001.

An expansion of the volume of the hydrogels in water, termed swelling, occurs due to their capacity to accommodate a substantial amount of water through hydrogen bonding.^64^ The swelling behavior of a hydrogel is essential for the absorption and delivery of drugs. The equilibrium swelling ratios (ESRs) of the CMC hydrogel and CRN@hydrogel were investigated in phosphate-buffered saline (PBS) solution (pH 7.4). As depicted in Fig. 4, both the CMC hydrogel and CRN@hydrogel underwent rapid swelling within the initial 30 min of immersion in PBS solution, followed by a deceleration in the swelling ratio. Equilibrium swelling was attained after 4 h of immersion. Both the CMC hydrogel and CRN@hydrogel exhibited significantly high swelling behaviors in PBS solution, with ESRs of 1627.88 ± 81.52% and 973.40 ± 45.87%, respectively. In comparison to the ESR of the CMC hydrogel, the ESR of the CRN@hydrogel was notably reduced. This could be attributed to the presence of CRN in the CRN@hydrogel, leading to a reduction in the content of the CMC hydrogel, consequently diminishing its water-absorbing capacity. Additionally, a substantial release of CRN from the surface and interior of the CMC hydrogel occurred into the PBS solution during the swelling process. The ESRs of the CMC hydrogel and CRN@hydrogel were considerably higher than those reported for some previously studied hydrogels (Table 1).^65–67^ The high swelling behavior observed in the prepared hydrogels may be ascribed to the intrinsic hydrophilic nature of CMC, facilitated by the hydrogen bonding interactions between the carboxylate groups and water molecules.^68^ Hydrogel materials exhibiting a high water-absorption capacity are conducive to the absorption of wound exudates.

**Table 1:** Equilibrium swelling ratios of some reported hydrogels

Studies	Hydrogel composition	Maximum ESR	Literature
Shen *et al.*	Gelatin methacryloyl (GelMA) hydrogel	31.69 ± 1.43%	65
Jin *et al.*	Gelatin/polyhexamethylenebiguanide (PHMB) hydrogel	315.92 ± 24.75%	66
Chuysinuan *et al.*	Fibroin-alginate hydrogel	325%	67

They can adhere to the wound surface, maintaining a conducive moist environment that is advantageous for wound healing.^69^ We also assessed the mass remaining ratio of the hydrogels after the swelling experiments (Fig. S3†). The results revealed that the mass remaining ratio of the CMC hydrogel was 96.06 ± 1.12%, significantly exceeding that of the CRN@hydrogel (67.06 ± 1.37%). The potential cause for the mass loss in the CMC hydrogel could be incomplete crosslinking, leading to the dissolution of uncrosslinked CMC in the PBS solution. In the case of the CRN@hydrogel, besides the incomplete crosslinking of the CMC hydrogel, a more substantial reason for the increased mass loss was the extensive release of CRN into the PBS solution during the swelling process.

### 2.4 *In vitro* assessment of cumulative CRN release from the CRN@hydrogel

**Fig. 5:**
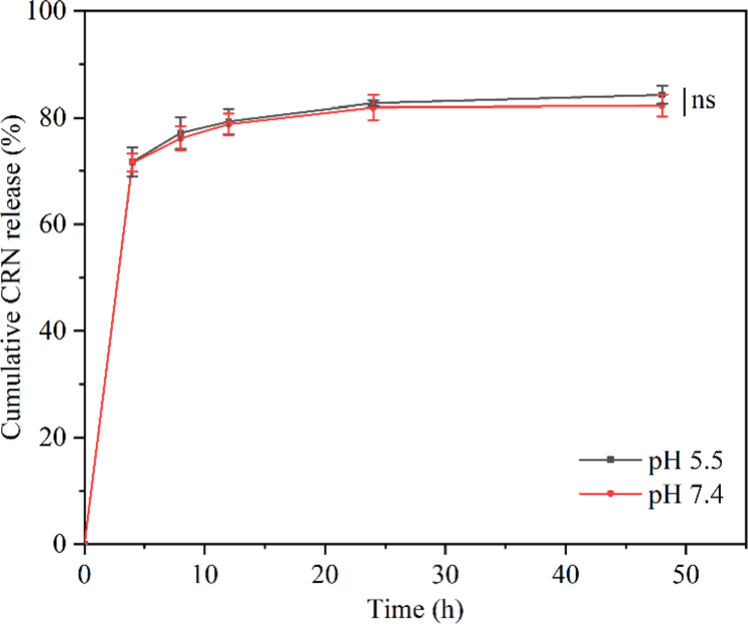
Cumulative CRN release from CRN@hydrogel in PBS solution (pH = 5.5 and 7.4). ns represents no significant difference.

Since inflammatory wound sites, such as diabetic wounds, are usually characterized by an acidic pH,^70^ we investigated the release properties of the CRN@hydrogel at pH 5.5 and pH 7.4 (Fig. 5). Under acidic conditions (pH 5.5), the maximum release of the CRN@hydrogel at 48 h was approximately 84.25%. Conversely, in a neutral environment (pH 7.4), the maximum release of the CRN@hydrogel at 48 h was approximately 82.25%. Furthermore, the cumulative release amounts of the CRN@hydrogel in pH 5.5 and 7.4 environments showed no statistically significant difference (*P* = 0.257). During the initial hours in the pH 5.5 and pH 7.4 environments, the cumulative CRN release percentage of the CRN@hydrogel significantly increased. This could likely primarily be attributed to the adsorption of a portion of CRN on the surface of the CMC hydrogel, allowing for a rapid release in the solution. As time progressed, there was a gradual slowdown in CRN release, possibly mainly due to the diffusion of CRN from the hydrogel matrix into the solution. Our experimental findings showed that the CRN@hydrogel could release CRN rapidly in a short time in both neutral and acidic environments, and more than 80% of the CRN could be released within 48 h. Foroushani *et al.* fabricated silk fibroin-chitosan-silver-curcumin nanofibers utilizing an electrospinning method and investigated their release behavior in PBS solutions at pH 5.4 and pH 7.4. The results indicated that the nanofibers released less than 60% of curcumin within 48 h in the various PBS solutions.^71^ Li *et al.* prepared a hydrogel loaded with metformin using hyaluronic acid and polyvinyl alcohol, and found that the hydrogel released less than 30% of the metformin in both pH 7.4 and 5.0 PBS solutions within 48 h.^72^ Compared to previous literature, the CRN@hydrogel exhibited the capability to achieve the rapid release of a greater quantity of CRN within 48 h, thereby enhancing its efficacy.

### 2.5 Cytocompatibility performance *in vitro*

**Fig. 6:**
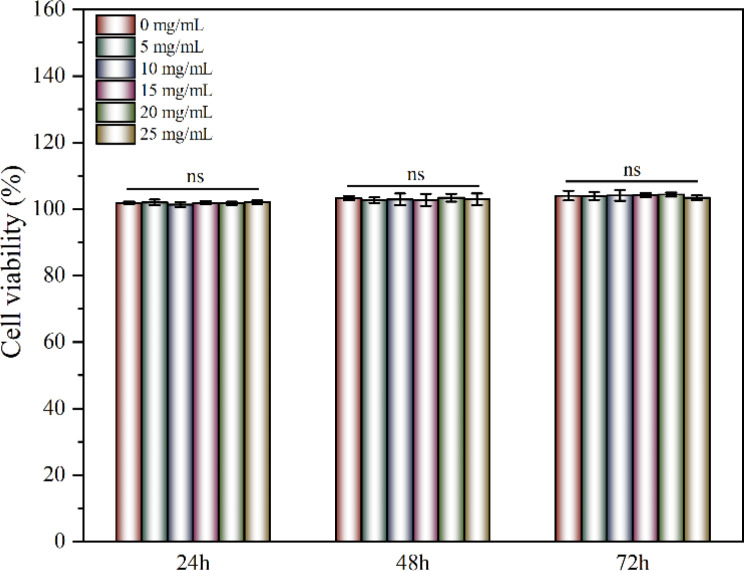
Cells viabilities of keratinocytes after cultured with different concentrations of CRN@hydrogel for 3 days. ns represents no significant difference.

Keratinocytes were cultured with the CRN@hydrogel at different concentrations and the Cell Counting Kit-8 (CCK-8) was used to detect the activity of the corresponding cells. When co-culturing the hydrogel extracts and keratinocytes separately for 24, 48, and 72 h, the cell viabilities remained largely unaffected by the varying concentrations of CRN treatment. The cell viabilities for all the samples exceeded 100% (Fig. 6). Previous work has shown that CMC hydrogels have good cytocompatibility.^37,73^ Ali *et al.* designed CMC hydrogels containing reduced graphene oxide, and the results of their cytocompatibility experiments showed that the cell survival rates of all the samples exceeded 90%.^37^ The cell survival rates of CMC-polyethylene glycol hydrogels prepared by Capanema *et al.* exceeded 95%.^73^ Our experimental results showed that the CRN@hydrogel had excellent cytocompatibility. Furthermore, we further investigated the blood compatibility of the CRN@hydrogel. As depicted in Fig. S4,† the red blood cells in the deionized water group were almost completely ruptured. However, upon the addition of varying concentrations of CRN@hydrogel, red blood cell rupture was relatively rare, with hemolysis ratios generally remaining below 2%. Biomaterials with a hemolysis ratio of less than 5% are acceptable as wound dressings.^74^ When the CRN concentration was less than 10 mg mL^−1^, the hemolysis ratio increased with the increase in CRN concentration. When the CRN concentration was between 10–20 mg mL^−1^, the hemolysis ratio decreased with the increase in CRN concentration. This phenomenon may be attributed to differential mechanisms of action exerted by the hydrogel samples on red blood cells at varying drug concentrations. Under low-concentration conditions, CRN may influence certain physiological processes of red blood cells, resulting in a decrease in their viability. Conversely, under high-concentration conditions, CRN may affect other physiological processes of red blood cells, thereby increasing their viability. The specific mechanisms underlying these observations remain unclear and require further experimentation for elucidation in future studies. These results indicated that the CRN@hydrogel will not cause damage to red blood cells and exhibited excellent blood compatibility. In conclusion, CRN@hydrogel had excellent cytocompatibility and can be safely used in subsequent *in vivo* experiments.

### 2.6 Wound-repair evaluation in an incision-wound rat model

In order to investigate the effect of the CRN@hydrogel on incision wounds, we created a rat incision-wound model. As shown in Fig. S5,† the incision-wound area of the rats in each group decreased with the extension of the treatment time. We assessed the average incision-wound-repair rates on postoperative days 3, 6, 9, and 12 for each group (Fig. S6†). On postoperative days 3, 6, 9, and 12, both the CRN@hydrogel and hydrogel groups exhibited significantly higher wound-repair rates compared to the control group. The wound-repair rate of the hydrogel group was higher than that of the control group on postoperative days 3, 6, 9, and 12. Furthermore, on postoperative days 3, 6, 10, and 12, the CRN@hydrogel group demonstrated significantly higher wound-repair rates compared to the hydrogel group. The differences in incision-wound-repair rates among the groups at each postoperative time point were statistically significant. On postoperative day 12, the incision wounds in the CRN@hydrogel group were almost completely repaired, and the scab formation at the incision site was completely removed. The hydrogel group retained some residual scabs, while the control group showed a greater amount of scab remnants. The results showed that the CRN@hydrogel group was superior to the hydrogel group in promoting incision-wound healing. Although the designed hydrogel could promote the healing of incision wounds, it lacked sufficient adhesive properties to facilitate rapid closure of the incision site in the initial postoperative period. Researchers have already explored the incorporation of highly adhesive substances into hydrogel designs to expedite the closure of incision wounds.^28,75^ In the work by Kang *et al.*, an injectable hydrogel bioadhesive with an on-demand removal trait was developed through the dynamic crosslinking of gelatin, tannic acid quinone, and borax. *In vivo* experimental results demonstrated that untreated incisions in rats can enlarge due to activity, hindering the wound-healing process, while wounds treated with the hydrogel exhibited proper closure. After 14 days postoperatively, wounds treated with both sutures and the hydrogel were nearly completely healed.^76^ In future research, the incorporation of highly adhesive substances into hydrogels can be explored for the design of biocompatible adhesives for wound closure. The aim is to expedite wound closure, minimize the risk of infection, and enhance the overall wound-healing process.

### 2.7 Wound-repair evaluation in a full-thickness-wound rat model

**Scheme 2:**
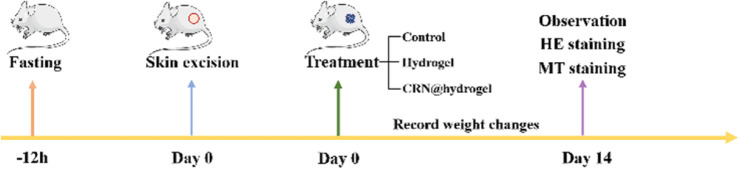
Schematic diagram of full-thickness-wound modeling and treatment process.

**Fig. 7:**
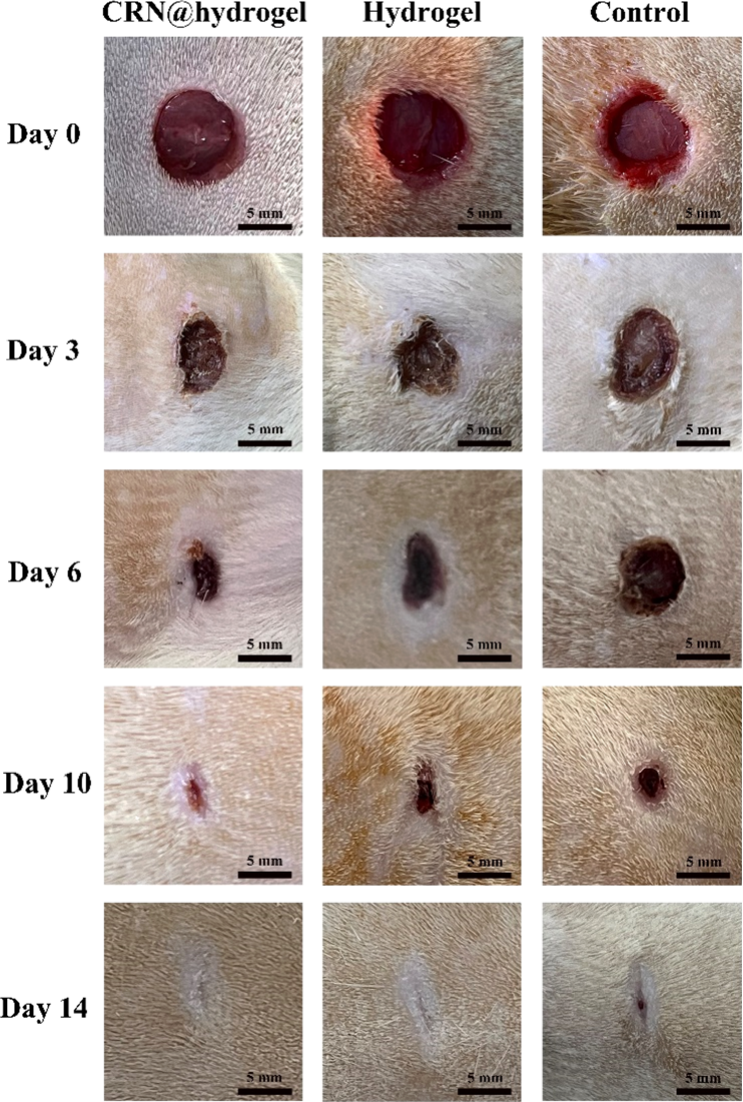
Wound-healing process of rats treated with CRN@hydrogel, hydrogel, and normal saline within 14 days in the full-thickness-wound model.

**Fig. 8:**
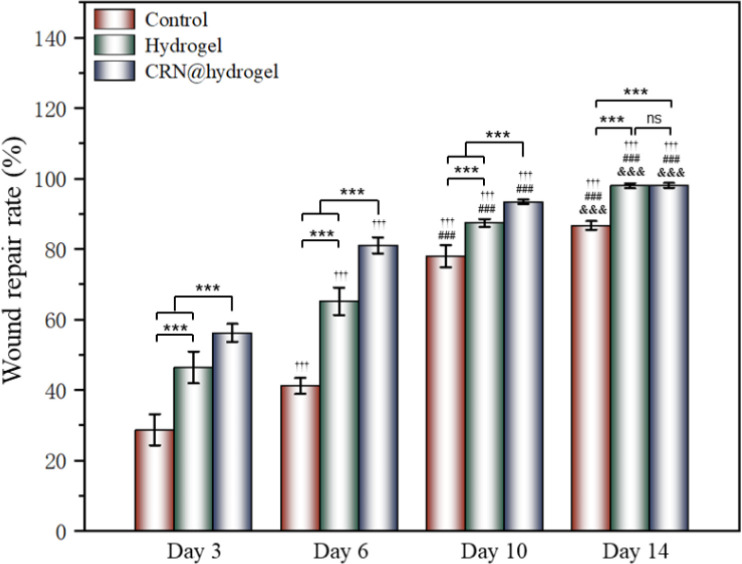
Average wound-closure rates of CRN@hydrogel, the hydrogel and control groups on postoperative days 3, 6, 10 and 14. ***P < 0.001, ††† represents a statistically significant difference (*p* < 0.001) when compared to the wound closure rate of the same group on the third day, ### represents a statistically significant difference (*p* < 0.001) when compared to the wound closure rate of the same group on the sixth day, &&& represents a statistically significant difference (*p* < 0.001) when compared to the wound closure rate of the same group on the tenth day, and ns represents no significant difference.

**Fig. 9:**
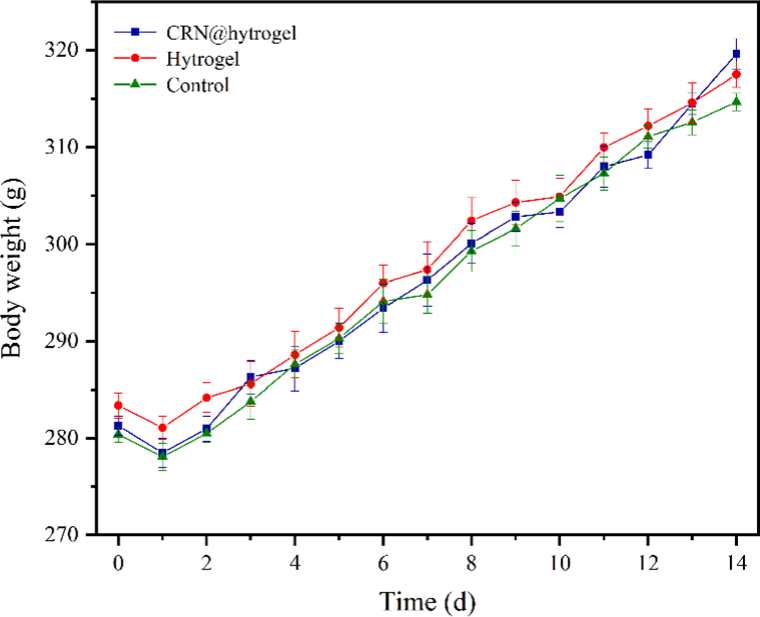
Changes in the body weight of Sprague-Dawley rats during the whole process.

To further assess the ability of the CRN@hydrogel to facilitate the wound-healing process, we performed wound-repair analysis using a full-thickness-wound rat model (Scheme 2). A full-thickness wound was generated in the rat by surgically excising the epidermis, dermis, and subcutaneous fat. This wound model was employed to simulate acute wound formation in clinical practice. As shown in Fig. 7, the wound area of the rats in each group decreased with the extension of the treatment time. We evaluated the average wound repair rates on postoperative days 3, 6, 10, and 14 for each group (Fig. 8). On postoperative days 3, 6, 10, and 14, the wound-repair rates were significantly increased in the CRN@hydrogel and hydrogel groups compared with the control group. Besides, the hydrogel group had higher wound-repair rates relative to the control group on postoperative days 3, 6, 10, and 14. The wound-repair rates of the CRN@hydrogel group were significantly higher than that of the hydrogel group on postoperative days 3, 6, and 10; however, there was no significant difference between the two groups on postoperative day 14. On postoperative day 14, the wounds in the CRN@hydrogel and hydrogel groups were almost completely repaired, with wound-repair rates of 98.06 ± 0.65% and 97.96 ± 0.68%, respectively. Statistical significances were observed in the differences in the wound-repair rates among the samples in each group across the various postoperative time points. In addition, we also measured the body weight of all the rats on each postoperative day (Fig. 9). On the first day after surgery, all the rats experienced body weight loss, which may be related to the reduced food and water intake caused by anesthesia, postoperative pain, and traumatic stress. Starting from postoperative day 2, the body weight of all the rats gradually increased. No significant differences were observed in the body weights of the rats between groups. The results showed that full-thickness skin excision only had a limited impact on rats in the short term after surgery, and this impact gradually disappeared over time. The above results indicated that the CRN@hydrogel and hydrogel groups could significantly improve wound healing compared with the control group, and the CRN@hydrogel group could accelerate wound healing relative to the hydrogel group. The hydrogels we designed demonstrated superior efficacy in the repair of full-thickness wounds compared to some previously reported hydrogels.^77,78^ Nooshabadi *et al.* developed a chitosan hydrogel loaded with exosomes for the repair of full-thickness wounds with a diameter of 7 mm on the dorsum of mice. The results indicated a wound-closure rate of 83.6 ± 4.9% on postoperative day 14.^77^ Wang *et al.* developed a hydrogel based on dopamine, gelatin, and polyacrylamide, loaded with doxycycline hydrochloride, for the repair of full-thickness wounds with a diameter of 7 mm on the dorsum of rats. The results revealed a wound-closure rate of 95.72% on postoperative day 14.^78^ CMC hydrogels contribute to wound healing by sustaining a moist environment in the surrounding wound area. This environment facilitates the proliferation and migration of fibroblasts and keratinocytes. Additionally, the maintained moisture enhances cell growth, enzymatic activity, growth factor function, and hormonal effects. Furthermore, these hydrogels exhibit advantageous features, including autolytic debridement of slough and necrotic tissue, while concurrently preventing bacterial growth.^32^ In addition, CMC hydrogels can activate macrophages and increase wound cytokine levels.^79^ Nagai *et al.* proposed the mechanistic role of CRN in wound healing, including inducing early effusion, promoting nucleic acid synthesis, and β-alanine-induced collagen synthesis. This contributes to granulation and expedites the healing process of wound tissue.^80^ Existing research has indicated that CRN can stimulate the production of growth factors, such as insulin-like growth factor 1 (IGF1), transforming growth factor-β (TGF-β), and stromal-derived factor 1 (SDF1), in wound sites. Furthermore, a notable elevation in the expression levels of the remodeling proteins collagen 1a and smooth muscle actin 1 (SMA1) has been observed in wounds subjected to CRN treatment.^48^ Incorporating CRN into the CMC hydrogel represents a promising approach that can enhance the therapeutic efficacy of the CMC hydrogel in the wound-healing process.

### 2.8 Histological analysis

**Fig. 10:**
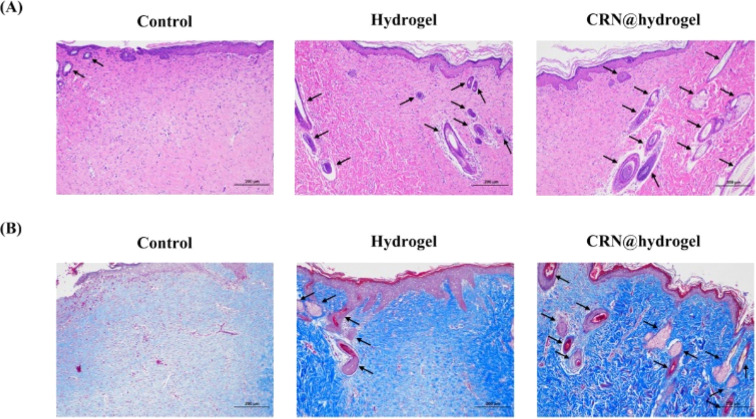
(A) Representative images of hematoxylin and eosin stained CRN@hydrogel, hydrogel, and control groups on postoperative day 14. (B) Representative images of the CRN@hydrogel, hydrogel, and control groups on postoperative day 14 stained with Masson's trichromatic stain. The black arrows indicate skin appendages.

In order to evaluate the healing effect of the CRN@hydrogel on the full-thickness wounds in the rats, hematoxylin and eosin (HE) staining was used to assess the histological changes at the wound site on postoperative day 14. The regeneration of epithelial structures, blood vessels, and hair follicles plays an active role in wound healing.^81^ Normal skin consists of three layers, namely (from top to bottom) the epidermis, dermis, and subcutaneous tissue.^82^ The epidermis is an epithelial tissue composed of multiple layers of keratinocytes, with the outermost layer predominantly consisting of keratinized cells, serving a protective function.^83,84^ The skin appendages encompass sweat glands, hair follicles and hair, sebaceous glands, and nails. Sweat glands are primarily distributed within the dermal layer. Hair follicles are located in the dermal layer, with hair extending through the dermis into the epidermal layer. Sebaceous glands are situated in the dermal layer, commonly associated with hair follicles.^83–85^ As shown in Fig. 10A and S7A,† the wounds did not heal completely in the control group. The epidermis and dermis layer could be observed, but the regeneration effect of the skin appendages was poor. The wounds in the CRN@hydrogel and hydrogel groups were repaired completely, and the newly formed epithelial structure had completely covered the wounds; in addition, the density of skin appendages in the CRN@hydrogel group was higher. Lin *et al.* developed a dermal ECM hydrogel encapsulating adipose-derived stem cells and endothelial progenitor cells for the repair of full-thickness wounds in rats. On postoperative day 14, the wounds treated with this hydrogel exhibited a limited formation of skin appendages, whereas our experiments revealed a significant increase in skin appendage formation. The application of hydrogels topically facilitated the repair of both the epidermis and dermis. This was achieved through the creation of a moist environment, which prevents secondary infections, fosters cellular proliferation and migration, and expedites wound epithelialization.^86^ Wound tissues treated with CRN exhibited enhanced granulation tissue formation, potentially attributed to an increased expression of growth factors and cytokines involved in wound healing.^48^

Collagen fiber is an important component of the dermal ECM, and the deposition of collagen fiber is conducive to wound healing.^87^ The important function of collagen fiber is to provide structural support and a living microenvironment for tissue cells.^88^ Collagen, as the principal structural protein in the skin, plays a vital role in the effective reconstruction of dermal tissue at wound sites.^89^ Scar formation is primarily attributed to an inadequate synthesis and deposition of collagen at the wound site, faulty fibroblast migration, and diminished formation of granulation tissue during the healing process.^90^ Masson's trichromatic (MT) staining can stain collagen fibers as blue. The more collagen fibers there are, the deeper the staining will be, which can be used to assess the degree of collagen fiber deposition.^91^ As shown in Fig. 10B and S7B,† on postoperative day 14, the MT staining results showed that a large number of collagen fibers were neatly arranged, evenly distributed, and dense in the CRN@hydrogel and hydrogel groups; in addition, the number of collagen fibers in the CRN@hydrogel group was higher and the staining was deeper. Although collagen fibers were also distributed in the control group, the arrangement was looser, the distribution was uneven, the staining was light, and the number was small. In comparison to certain hydrogel materials previously reported,^78,92,93^ our MT staining results exhibited similar outcomes. It has been proved that a large amount of collagen accumulates in wound tissue after CRN treatment.^48^ These results indicated that the CRN@hydrogel can better promote collagen deposition and arrangement, and further accelerate the wound-healing process.

## 3. Conclusions

In this study, we co-assembled CRN with CMC hydrogel to fabricate a novel CRN@hydrogel, hoping to give full play to the advantages of both. *In vitro* experiments confirmed that the CRN@hydrogel could release over 80% of the drug within 48 h, as well as demonstrated its favorable cytocompatibility and blood compatibility; thus establishing its applicability for safe utilization in clinical practice. By using the rat model, we found that the CRN@hydrogel could promote and accelerate wound healing more effectively. These results indicate that this novel hydrogel has potential to serve as an efficient therapeutic strategy for wound treatment.

## 4. Materials and methods

### 4.1 Materials

All the chemicals utilized in this study were selected for their superior purity or higher. If not explicitly mentioned, no further purification was employed for the chemicals. Carboxymethyl cellulose (CMC, MW = 700 000), sodium hydroxide (NaOH, 99%), epichlorohydrin (Analytical reagent), and ethanol (Analytical reagent) were purchased from McLean (China). l-Carnosine (CRN, 99%) was purchased from Sigma-Aldrich/Merck (Germany). Hematoxylin dye, eosin dye, chloral hydrate (99%), methanol (≥99.7%), ammonium hydroxide (analytical reagent), dimethylbenzene (analytical reagent), and neutral gum were purchased from Sinopharm Chemical Reagent (China). Masson's trichromatic (MT) staining kit was purchased from Solarbio (China). The Cell Counting Kit-8 (CCK-8) kit was purchased from Beyotime Biotechnology (China).

### 4.2 Synthesis of the CMC hydrogel and CRN@hydrogel

First, 3 g of CMC was dissolved in 3% W/V NaOH solution in a three-neck round-bottom flask and stirred for 30 min to obtain a viscous mixture. Then 6 mL of epichlorohydrin was added and stirred for 2 h. The mixture was poured into a glass culture dish and heated in an oven at 80 °C for 2 h. The prepolymer was collected and washed several times, and put in the oven for drying at 50 °C. The dried gel was collected and soaked in water for 12 h. Finally, the samples were freeze-dried to obtain the CMC hydrogel. Next, 25 mg mL^−1^ of CRN solution was prepared,^48^ and then hydrogel samples were synthesized with different ratios by mixing 5, 6, and 7 mg of CMC hydrogel separately with 200 μL of CRN solution in test tubes. The CRN@hydrogel was formed after a period of time in a 60 °C water bath. The gelation outcome of these samples was assessed through tube inversion experiments. After water bathing, the CRN@hydrogel was positioned upright and allowed to cool at room temperature for 10 min before slowly inverting the test tube. The observation of hydrogel slippage upon inversion was considered indicative of poor gelation, rendering it unsuitable for subsequent experiments.

### 4.3 Morphology of the CMC hydrogel

The freeze-dried CMC hydrogel was cut with a blade to obtain a cross-section, and then the hydrogel was sprayed with gold (15 mA, 90 s). The surface morphology of the hydrogel sample was observed by SEM (S4800, Hitachi) with a secondary electron detector, operated at an accelerating voltage of 3.0 kV. Utilizing ImageJ software (National Institutes of Health, Bethesda, MD, USA), the pore size of the hydrogel was determined based on three images of the hydrogel.

### 4.4 FTIR spectroscopy

Initially, 100 mg of potassium bromide powder was placed in a mortar, and grinding was performed clockwise for 3–5 rotations. Subsequently, 1 mg of lyophilized hydrogel sample or CRN powder was added to the potassium bromide powder, and the mixture was homogenized through grinding. The resulting mixture was then subjected to rapid drying in an infrared dryer. Then, a transparent tablet was formed by pressing the powder inside the tablet press. FTIR spectra of the CMC hydrogel, CRN, and CRN@hydrogel were recorded in the range of 4000–500 cm^−1^ by employing a Nicolet is50 FTIR spectrometer (Thermo Scientific Instrument). During the infrared measurements, a blank potassium bromide was utilized as a background, with a resolution exceeding 0.09 cm^−1^. Each sample was scanned 32 times, and the spectral data were analyzed and plotted using Origin (ver. 2021; OriginLab., USA) after the completion of data acquisition.

### 4.5 Rheological property

The rheological tests of the CMC hydrogel were carried out using a TA rheometer (HR-1). The CMC hydrogel sample was prepared into circular disks with a diameter of 8 mm and a thickness of 1 mm. Parallel plates with a diameter of 8 mm were chosen, and the gap between the parallel plates was set at 0.5 mm for the hydrogel testing. During the testing procedure, excess gel was removed, and a thin layer of petroleum jelly was applied to seal the edges of the hydrogel to prevent moisture evaporation during testing. The testing temperature was set at 25 °C. Initially, a dynamic frequency sweep was conducted with a fixed strain of 1% to examine the changes in *G*′ and *G*′′ of the hydrogel within the frequency range of 0.1–100 rad s^−1^. Subsequently, a dynamic strain sweep was performed at a fixed frequency of 1 Hz to evaluate the modulus variation of the hydrogel within the strain range of 10–100%, aiming to assess the stability of the hydrogel's internal three-dimensional structure.

### 4.6 Swelling test

The ESRs of the CMC hydrogel and CRN@hydrogel were determined by swelling tests. The freeze-dried hydrogel was accurately weighed (Wi) and immersed in a PBS solution with pH 7.4. At predefined time intervals (10 min, 30 min, 1 h, 2 h, 4 h, 8 h, 12 h, and 24 h), the hydrogel was retrieved, and excess surface water was removed using filter paper. Subsequently, the hydrogel was reweighed (*W*_s_). The ESR of the hydrogel was calculated using the formula: ESR = (*W*_s_ − *W*_i_)/*W*_i_ × 100%. The sample subjected to the swelling test was freeze-dried, and the weight was recorded again (*W*_d_). The mass remaining ratio was calculated using the formula: *W*_d_/*W*_i_ × 100%. All the experiments were conducted in triplicate.

### 4.7 *In vitro* assessment of cumulative CRN release from the CRN@hydrogel

The concentration of CRN was measured using an ultraviolet-visible spectrophotometer (UV-2600i, Shimadzu Instruments Manufacturing Co., Ltd). The fundamental structure of the ultraviolet-visible spectrophotometer consisted of five main components: a light source, monochromator, sample chamber, detector, and measurement system. The fundamental principle underlying the measurement of absorbance at different wavelengths involves the interaction between light and the sample. As light passes through the sample, certain wavelengths are absorbed by the sample molecules. The absorbance at a specific wavelength is directly proportional to the concentration of absorbing species in the sample.

First, we prepared CRN solutions with concentrations of 0.15, 0.3, 0.45, 0.6, 0.75, and 0.9 mg mL^−1^ using PBS solution (pH 7.4). Before conducting ultraviolet spectroscopy analysis on the samples, the absorbance of PBS at the designated wavelength was calibrated as a reference to eliminate the potential influence of PBS. Ultraviolet spectroscopy was then performed on each solution to determine the wavelength range of the maximum absorption peak based on the absorbance peak values at different concentrations. Each concentration of CRN solution exhibited a maximum absorption peak at the wavelength of 258 nm (Fig. S8A†). The analysis of the relationship between different CRN concentrations and their respective absorbances at 258 nm was conducted using Origin, leading to the derivation of the CRN standard curve. As depicted in Fig. S8B†: *y* = 1.689*x* + 0.0502, *R*^2^ = 0.9917, indicating a robust linear correlation. This enabled the conversion of the absorbance readings in the ultraviolet spectroscopy to concentrations. To investigate whether the degrading hydrogel in PBS affects the absorbance of CRN, we immersed the CMC hydrogel into a 15 mL PBS solution. After 48 h, 2 mL of the supernatant was collected for ultraviolet analysis. The results revealed no peak at 258 nm in the supernatant, suggesting that degradation of the hydrogel did not affect the absorbance of CRN (Fig. S9†). Finally, an investigation was conducted to explore the release performance of CRN from the CRN@hydrogel. Here, 7 mg of CMC hydrogel was added to a 200 μL solution of CRN with a concentration of 25 mg mL^−1^. Once gelation occurred, the CRN@hydrogel was placed in 15 mL of PBS solution (pH 5.5, 7.4). At the appointed point in time (0, 4, 8, 12, 24, 48 h), 2 mL of the supernatant was collected from each sample for ultraviolet analysis, and an immediate replenishment of 2 mL of PBS solution was made. The cumulative release amount of CRN was calculated using the following formula: cumulative release% = (15*C*_*n*_ + 2∑*C*_*n*−1_)/*M*_0_ × 100% (C_*n*_ and *C*_*n*−1_ are the concentrations of CRN (mg mL^−1^) in the releasing medium after *n* and *n*−1 withdrawing steps, respectively; *n* is the number of withdrawing steps of releasing media; *M*_0_ is the amount of CRN (mg) loaded in the samples). All the experiments were performed in triplicate.

### 4.8 Evaluation of the cytocompatibility *in vitro*

To verify the cytocompatibility of the CRN@hydrogel, human keratinocytes were selected for *in vitro* experiments. The keratinocytes were sourced from primary cells obtained from the Institute of Basic Medical Sciences, Chinese Academy of Medical Sciences. According to the international biomaterial standards (ISO10993-5), the hydrogel extraction method was recommended and optional for cytocompatibility experiments. We also referred to the previously published literature, where a growing number of researchers have used hydrogel extracts to investigate the cytocompatibility of various hydrogel dressings such as catechol-functionalized quaternized chitosan/dibenzaldehyde-terminated poly(ethylene glycol) hydrogel, carboxymethyl chitosan/tannic acid hydrogel, and quaternary ammonium chitosan/tannic acid hydrogel).^7,94,95^ Therefore, we chose the hydrogel extraction method to explore the cytocompatibility. To prepare the hydrogels, 200 μL of CRN solution at various concentrations (0, 5, 10, 15, 20, 25 mg mL^−1^) were mixed with 7 mg of CMC. The resulting hydrogels were sterilized by exposure to ultraviolet light (wavelength 254 nm) for 24 h on a clean bench (JOYN-CJ-1G, Shanghai Joyn Electronic CO., Ltd). Subsequently, the hydrogel was immersed in 200 μL of cell culture medium for 24 h, maintained at a constant temperature of 37 °C, thereby obtaining the hydrogel extract. The hydrogel extract was aseptically filtered through a needle-type filter and stored at 4 °C for future use. The cell culture medium chosen was Dulbecco's modified eagle's medium (DMEM)/F-12 supplemented with 10% fetal bovine serum (FBS) and 1% penicillin-streptomycin. The cells were incubated and expanded in a CO_2_ cell culture incubator at 37 °C. Passaging of cells was conducted using enzymatic digestion, and cells were passaged up to the 3rd to 5th generation for formal experiments. Cell seeding was performed in a 96-well plate at a density of 1 × 10^4^ cells per mL, with 100 μL of culture medium added. After removing the culture medium, 100 μL of the extraction solution was added to each well, and the plates were further incubated for 24, 48, and 72 h in the cell culture incubator. At each time point, the 96-well plate was retrieved, and 10 μL of CCK-8 working solution was added to each well, followed by a 2 h incubation period. Absorbance was measured at 450 nm using a microplate reader (infinite 200Pro). The negative control group consisted of keratinocytes, culture medium, and CCK-8 working solution, while the blank control group contained culture medium and CCK-8 working solution. The entire experiment was replicated three times. According to previous literature,^96–98^ the cell viability was calculated using the following formula: cell viability% = (OD_S_ − OD_B_)/(OD_N_ − OD_B_) × 100% where OD_S_, OD_B_, and OD_N_ are the absorbance of the sample, the blank control, and the negative control at 450 nm, respectively.

Furthermore, we conducted *in vitro* hemolysis experiments using Sprague-Dawley (SD) rat red blood cells to assess the hemolysis rate of the CRN@hydrogel. To achieve this, 1 mL of CRN solution at various concentrations (0, 5, 10, 15, 20, 25 mg mL^−1^) was mixed with 35 mg of CMC to prepare the hydrogels. The resulting hydrogels were sterilized by exposure to ultraviolet light (wavelength 254 nm) for 24 h on a clean bench. The hydrogels were then individually added to 1 mL of normal saline for 24 h, maintained at a constant temperature of 37 °C, thereby obtaining the hydrogel extracts. Subsequently, fresh red blood cells were collected from SD rats using EDTA anticoagulant tubes. After centrifugation at 3000 rpm for 10 min, the supernatant was removed. The red blood cells were washed with normal saline and this process was repeated three times. The collected red blood cells were suspended in normal saline to create a 2% volume fraction suspension. Finally, 1 mL of the red blood cell suspension was added to a centrifuge tube along with 1 mL of the hydrogel extract, and the mixture was incubated at 37 °C for 60 min. Next, 100 μL of the supernatant was transferred to a 96-well culture plate, and the absorbance was measured at 542 nm. Deionized water served as the positive control, and normal saline served as the negative control. The entire experiment was replicated three times. The hemolysis ratio was calculated using the following formula: hemolysis ratio% = (OD_S_ − OD_N_)/(OD_P_ − OD_N_) × 100% where OD_S_, OD_N_, and OD_P_ are the absorbance of the sample, the negative control, and the positive control at 542 nm, respectively.

### 4.9 Wound repair evaluation in incision-wound and full-thickness-wound experiments

Our animal experiments were approved by the Animal Center of Anhui Medical University Ethical Committee (LLSC 20201145). Experimental rats were obtained from the Experimental Animal Center of Anhui Medical University. All the experimental animals were individually housed in separate ventilated isolation cages, maintaining the temperature within the cages at 23 ± 2 °C and the relative humidity at 55–60%. The room environment was kept quiet with a 12 h light–dark cycle to provide suitable living conditions. Clean bedding was provided to maintain housing hygiene. Regular cleaning and disinfection of housing facilities were performed to prevent disease transmission. All the experimental animals were granted unrestricted access to standard maintenance feed and purified water, and the experiments were conducted after a one-week adaptation period. Routine health checks, including monitoring the body weight, behavior, and general health status of the rats, were conducted. During and after surgical procedures, the pain levels of the experimental animals were monitored and evaluated, with appropriate anesthesia and analgesia administered when necessary. This practice ensured sufficient pain relief for the experimental rats and allowed for adjustments as needed. A 12 h fasting period was implemented for all the rats prior to the surgical procedure. All the rats were anesthetized using sodium pentobarbital (40 mg kg^−1^) *via* intraperitoneal injection. The dorsal hair of the rats was shaved using an electric shaver. The inclusion criteria for the experimental rats were: (1) SD rats (male, aged 6–8 weeks, weighing 260 ± 20 g); (2) healthy rats, without any disease or infection; (3) in the establishment of the incision-wound experimental model, a sterile scalpel was employed to create a 1 cm incision on the dorsal skin of the rats. The incision was meticulously performed to avoid damaging the muscles and fascia, followed by applying pressure to the incision until hemostasis was achieved. (4) In the establishment of the full-thickness-wound experimental model, a hole punch was utilized to create the circular wound (8 mm × 8 mm)^99^ on the dorsal skin of the rats. Subsequently, the wounds were compressed to ensure hemostasis without any bleeding. The exclusion criteria were: (1) unhealthy rats with any disease or infection; (2) rats that died unexpectedly due to anesthesia during operation; (3) injury to muscles or fascia during surgery leading to unstoppable bleeding on the wound; (4) secondary injury to the back wound due to tearing and other reasons. Using the random number table method, 18 rats were randomly divided into three groups: one negative control group treated with normal saline (control group), one pure CMC hydrogel treatment group (hydrogel group), and one CRN-loaded CMC hydrogel treatment group (CRN@hydrogel group). To prepare the CMC hydrogel and CRN@hydrogel, 200 μL of distilled water and 200 μL of a 25 mg mL^−1^ CRN solution were individually mixed with 7 mg of CMC. The prepared normal saline, CMC hydrogel, and CRN@hydrogel underwent 24 h of ultraviolet irradiation at a wavelength of 254 nm for sterilization. After incision-wound modeling, 50 μL of normal saline, CMC hydrogel (containing 1.75 mg CMC), and CRN@hydrogel (containing 1.75 mg CMC and 1.25 mg CRN) were applied to the wound correspondingly, with replacement every 3 days. After full-thickness-wound modeling, 100 μL of normal saline, CMC hydrogel (containing 3.5 mg CMC), and CRN@hydrogel (containing 3.5 mg CMC and 2.5 mg CRN) were applied to the wound correspondingly, with replacement every 3 days. At postoperative specific time points, high-quality photos of the wounds on the back of rats were taken under anesthesia and measured by Image J software (National Institutes of Health, Bethesda, MD, USA). Based on the work of Abbasi *et al.*,^100^ the wound-repair rate was calculated using the following formula: wound-repair rate% = (*A*_0_ − *A*_*t*_)/*A*_0_ × 100% (where *A*_0_ is the initial area of the wound, and *A*_*t*_ is the wound area on day *t*). On each postoperative day, all rats were weighed using an electronic scale. During the experiment, the information of the experiment was not clear to both the experimental observer and the data analyst.

### 4.10 Histological analysis

On the 14th day after full-thickness skin excision, the wound samples were collected after the rats were humanely sacrificed. The new granulation tissue of the wound and the surrounding normal tissue were cut together. Then, the wound samples were fixed with 4% paraformaldehyde, and embedded in paraffin. The transverse sections (4 μm) of the wound samples were placed on glass slides for histological examination. Two sections were prepared for each wound sample, one for HE staining and one for MT staining. The prepared sections were dried in an oven at 60 °C. The wound healing was observed by HE staining, and the collagen fiber deposition was observed by MT staining.

For HE staining, the paraffin sections were immersed in dimethylbenzene for 20 min and repeated twice. Subsequently, the sections were immersed in anhydrous ethanol for 5 min and repeated twice. The sections were then transferred to 75% ethanol for 5 min and subsequently rinsed with water. The sections were stained with hematoxylin solution for 0.5–1 min. Subsequently, the sections were differentiated with 1% hydrochloric acid and alcohol for several seconds, and then treated with 1% aqueous ammonia solution for 1 min. Eosin dye solution was applied for several seconds, and the sections were subsequently immersed in varying ethanol concentrations to facilitate dehydration. The sections were fully immersed in dimethylbenzene for dehydration until they became transparent. Finally, the sections were sealed with neutral gum. Imaging of the sections was carried out using DS-Ri2 image acquisition system (NIKON).

For MT staining, the paraffin sections were immersed in dimethylbenzene for 20 min and repeated twice. Subsequently, the sections were immersed in anhydrous ethanol for 5 min and repeated twice. The sections were then transferred to 75% ethanol for 5 min and subsequently rinsed with water. The sections were stained with freshly prepared Weigert iron hematoxylin in a wet box protected from light for 10 min. Differentiation was performed using 1% hydrochloric acid alcohol for 10 s. The sections were treated with Masson blue solution in a wet box protected from light for 5 min and washed with distilled water for 1 min. Ponceau S Stain Reagent was used to stain for 10 min in a wet box protected from light, and 0.2% glacial acetic acid working solution was used to wash the sections for 1 min. Subsequently, the sections were treated with phosphomolybdic acid working solution for 5 min and washed with 0.2% glacial acetic acid working solution for 1 min. Aniline blue was used to stain the sections for 2 min in a wet box protected from light, and 0.2% glacial acetic acid working solution was used to wash the sections for 1 min. Dehydration was carried out in different concentrations of ethanol. The sections were fully immersed in dimethylbenzene for dehydration until they became transparent. Finally, the sections were sealed with neutral gum. Imaging of the sections was carried out using the DS-Ri2 image acquisition system (NIKON).

### 4.12 Statistical analysis

Statistical analysis was conducted using SPSS (ver. 27.0; SPSS Inc., USA). All data were expressed as the mean ± standard deviations. The results between groups were compared by one-way ANOVA or Student's *t*-test. The difference was considered statistically significant at the values of *P < 0.05, **P < 0.01 and ***P < 0.001.

## Data availability

The data underlying this article will be shared on reasonable request to the corresponding author.

## Author contributions

Wei Zhang: conceptualization, data curation, investigation, methodology, writing – original draft; Xinyi Li: data curation, investigation, methodology, writing – original draft; Wenjian Chen: experiment guidance and english grammar; Xiaoyi Huang: data curation, investigation, methodology, writing – original draft; Tianfeng Hua: data curation, investigation; Jinpeng Hu: investigation, methodology; Jing Zhu: conceptualization; Sheng Ye: conceptualization, funding acquisition, supervision; Xiaojing Li: conceptualization, funding acquisition, supervision.

## Conflicts of interest

The authors declare no conflict of interest.

## Supplementary Material

RA-014-D4RA00135D-s001
