# Supplementary material for: l-Carnosine loaded on carboxymethyl cellulose hydrogels for promoting wound healing
Source: RSC Adv. 2024 Jun 7;14(26):18317–29. doi: 10.1039/d4ra00135d (PMC11163232; doi:10.1039/d4ra00135d)
Supplement: RA-014-D4RA00135D-s001 [file RA-014-D4RA00135D-s001.pdf]

Electronic Supplementary Material (ESI) for RSC Advances.
This journal is © The Royal Society of Chemistry 2024

Supporting Information

L-carnosine Loaded on Carboxymethyl Cellulose Hydrogels Promoting Wound Healing

Wei Zhang,[a,†] Xinyi Li,[a,†] Wenjian Chen, [d,†] Xiaoyi Huang,[b,†] Tianfeng Hua,[c] Jinpeng Hu,[a] Jing Zhu,[b] Sheng Ye,[b,*] Xiaojing Li[a,*]

[a] Department of Plastic Surgery, The First Affiliated Hospital of Anhui Medical University, Hefei 230022, Anhui, China.

[b] School of Materials and Chemistry, Anhui Agricultural University, Hefei, Anhui 230036, China.

[c] Department of Emergency Surgery & The 2nd Department of Intensive Care Unit, the Second Hospital of Anhui Medical University, Hefei, Anhui 230001, China.

[d] Department of Orthopaedics, Anhui Provincial Children's Hospital, Hefei, Anhui 230022, China

* Correspondence: sye503@ahau.edu.cn (S.Y.); ay_lxj@yahoo.com (X.L.)

† These authors contributed equally to this work.

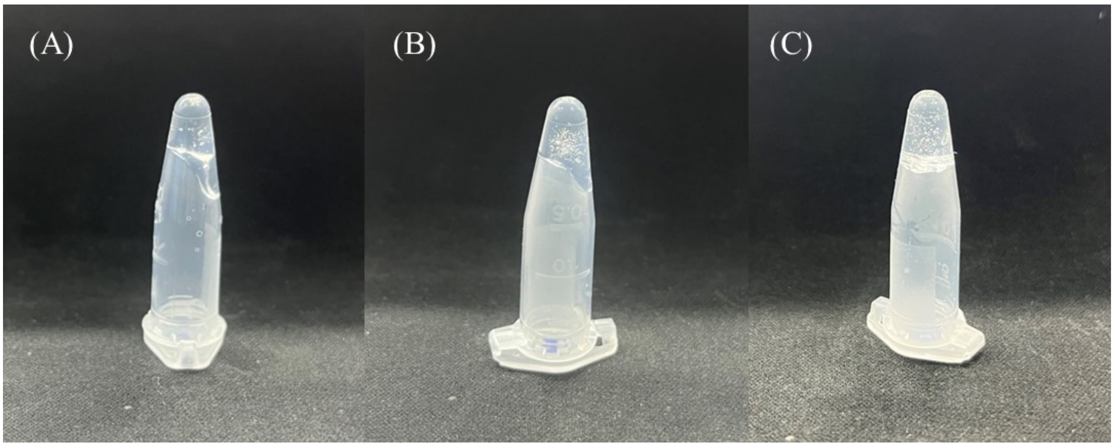

Figure S1. Hydrogel samples with different ratios by mixing (A) 5 mg, (B) 6 mg, and (C) 7 mg of CMC hydrogel separately with 200 µL of CRN solution in test tubes.

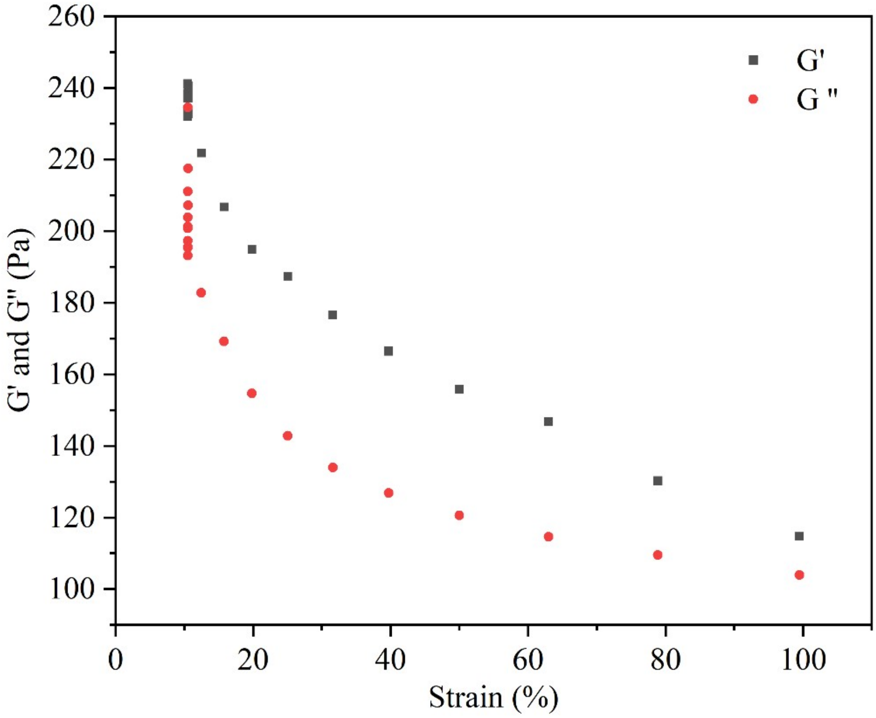

Figure S2. Frequency sweep test of the CMC hydrogel.

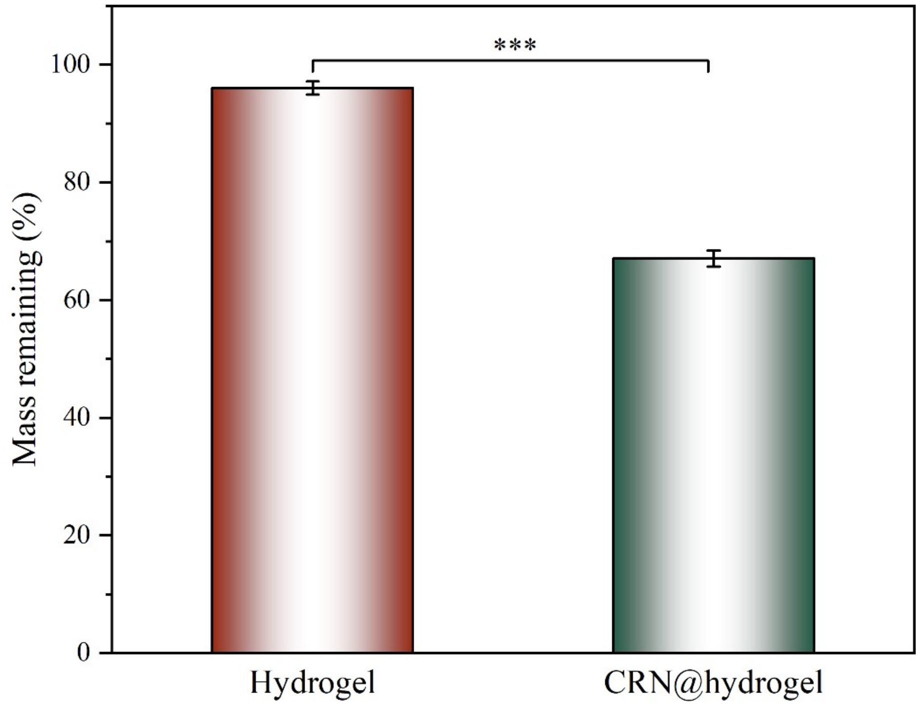

Figure S3. The mass remaining ratios of CMC hydrogel and CRN@hydrogel after swelling experiments. ***P < 0.001.

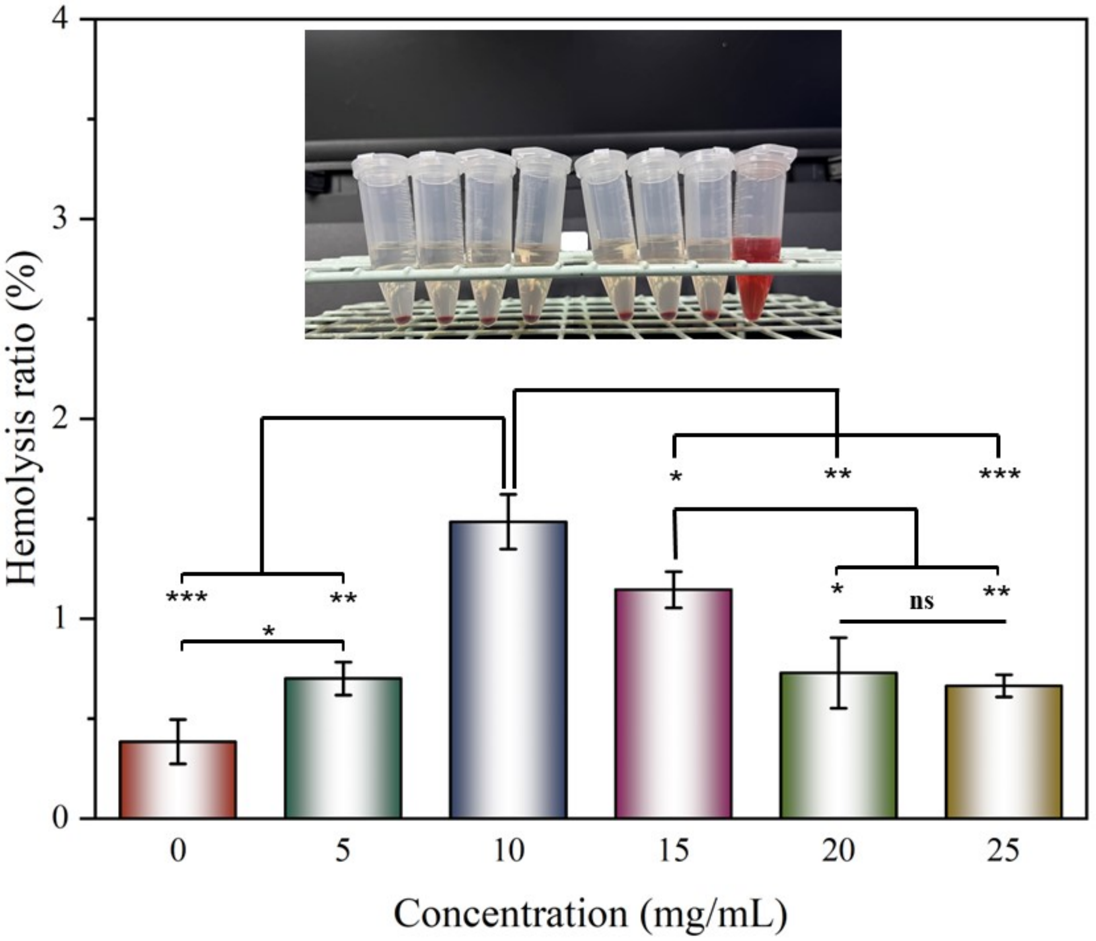

Figure S4. Hemolysis ratio of red blood cells incubated with CRN@hydrogel and the photographs of red blood cells after centrifugation in set. *P < 0.05, **P < 0.01 and ***P < 0.001. ns represents no significant difference.

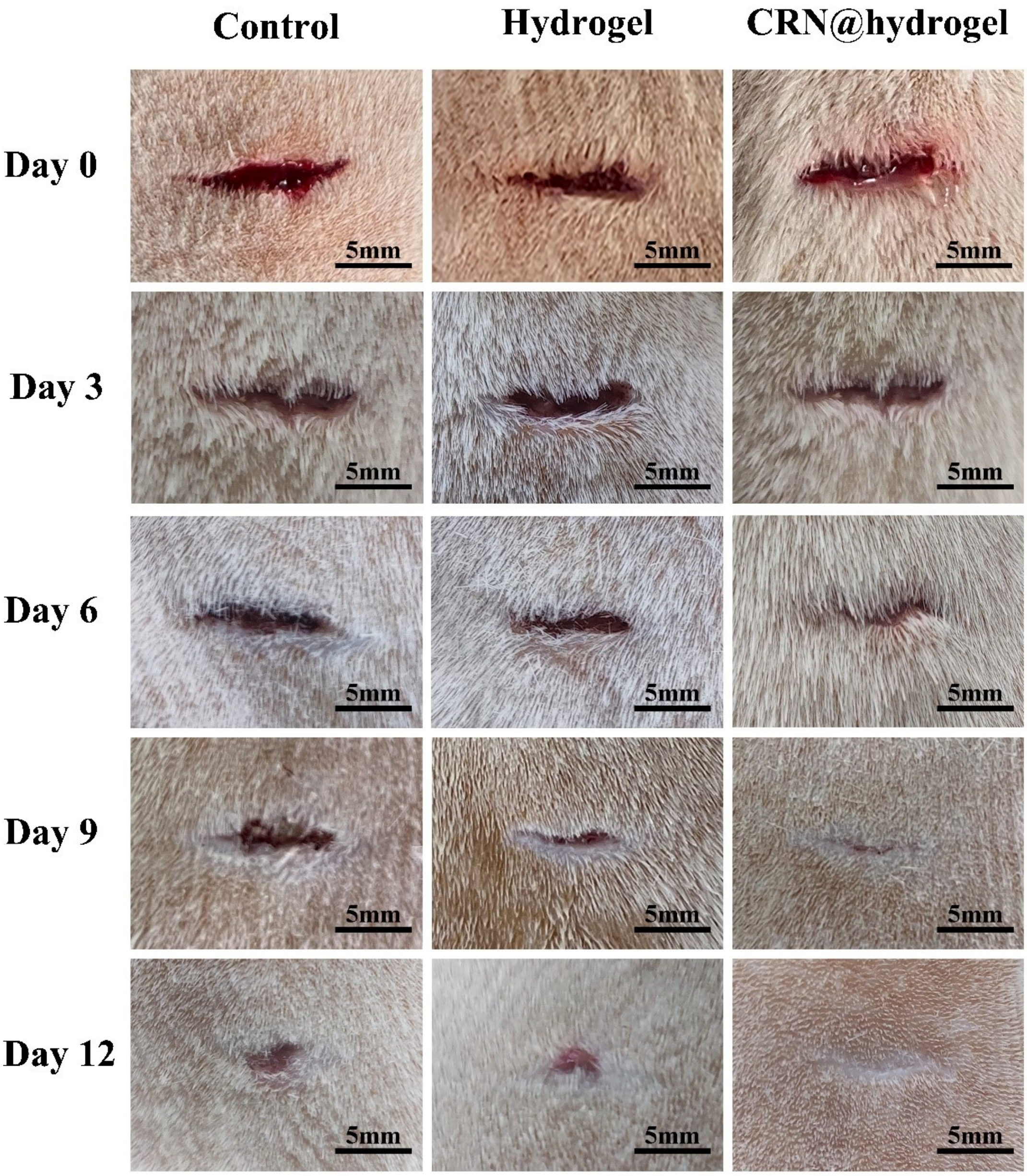

Figure S5. Wound healing process of treated rats with normal saline, hydrogel and CRN@hydrogel within 12 days of the incision model.

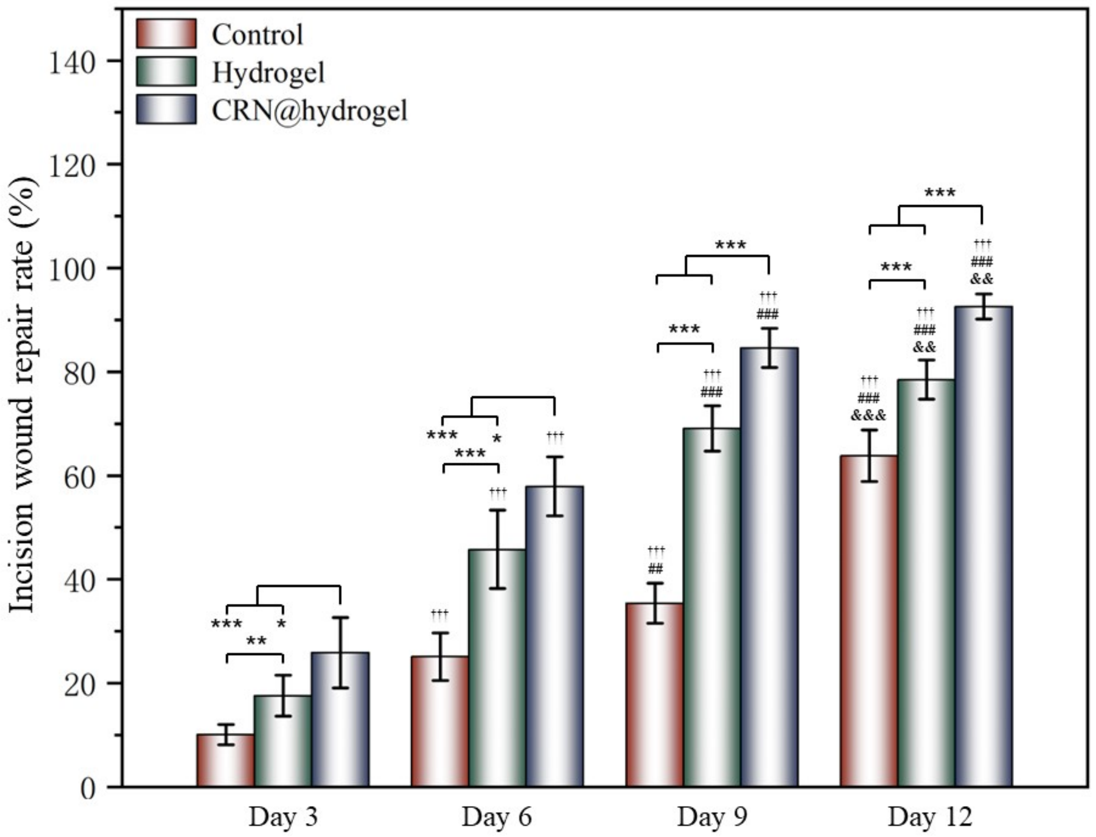

Figure S6. The incision wound closure rates of CRN@hydrogel, hydrogel and control groups on postoperative days 3, 6, 9 and 12. ***P < 0.001, ††† represents a statistically significant difference (p < 0.001) when compared to the wound closure rate of the same group on the third day, ### and ## represent statistically significant differences (p < 0.001 and p < 0.01 , respectively) when compared to the wound closure rate of the same group on the sixth day, &&& and && represent statistically significant differences (p < 0.001 and p < 0.01 , respectively) when compared to the wound closure rate of the same group on the ninth day.

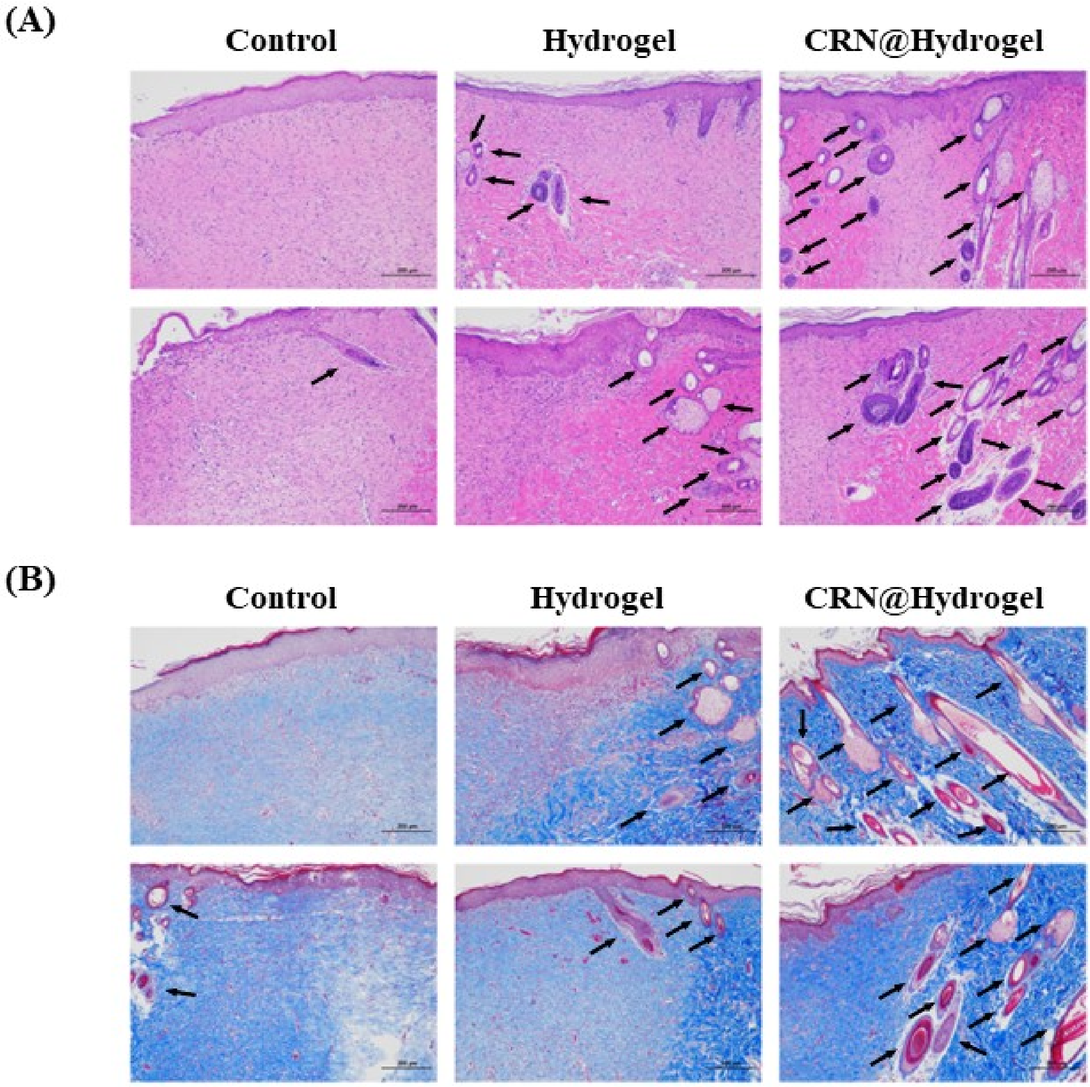

Figure S7. (A) Representative images of hematoxylin and eosin staining of CRN@hydrogel, hydrogel and control groups on postoperative day 14. (B) Representative images of Masson's trichromatic staining of CRN@hydrogel, hydrogel and control groups on postoperative day 14. The black arrows indicate skin appendages.

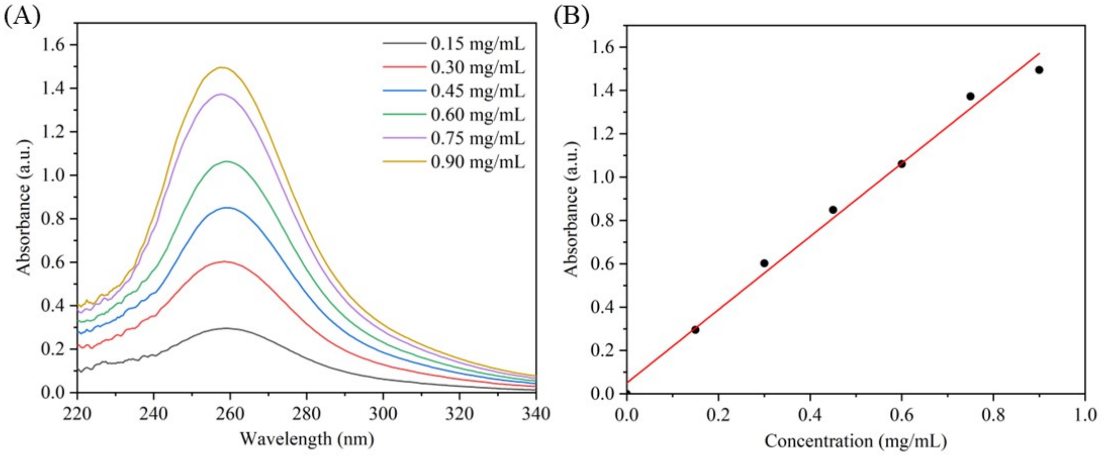

Figure S8. (A) Absorption curves of different concentrations of CRN. (B) Drawing of the standard curve of CRN.

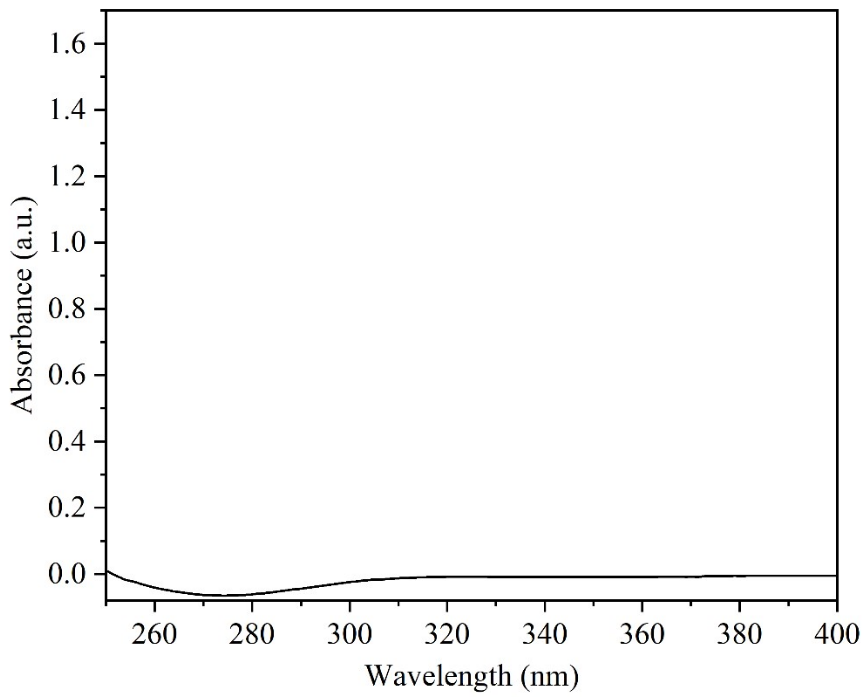

Figure S9. Ultraviolet analysis of the degradation product of the CMC hydrogel.
